# Supplementary material for: Diagnostic classification based on DNA methylation profiles using sequential machine learning approaches
Source: PLoS One. 2024 Sep 6;19(9):e0307912. doi: 10.1371/journal.pone.0307912 (PMC11379195; doi:10.1371/journal.pone.0307912)
Supplement: S1 Fig — (DOCX) [file pone.0307912.s001.docx]

**Supporting information for: Diagnostic classification based on DNA methylation profiles using sequential machine learning approaches.**

**Wojewodzic, M. W.** ^1,2,3*^, **Lavender, J. P.** ^4^

^1^ Cancer Registry of Norway, Norwegian Institute of Public Health, Oslo, Norway, ORCID: 0000-0003-2501-5201

^2^ Chemical Toxicology, Norwegian Institute of Public Health, Oslo, Norway,

^3^University of Birmingham, Birmingham, United Kingdom,

^4^ ORCID. 0000-0002-2052-7702

^*^Corresponding author: Marcin.Wojewodzic@kreftregisteret.no

**S1** **Fig** Normalization by GDC histograms (normal samples are colored blue and cancer red). 4 moments are indicated


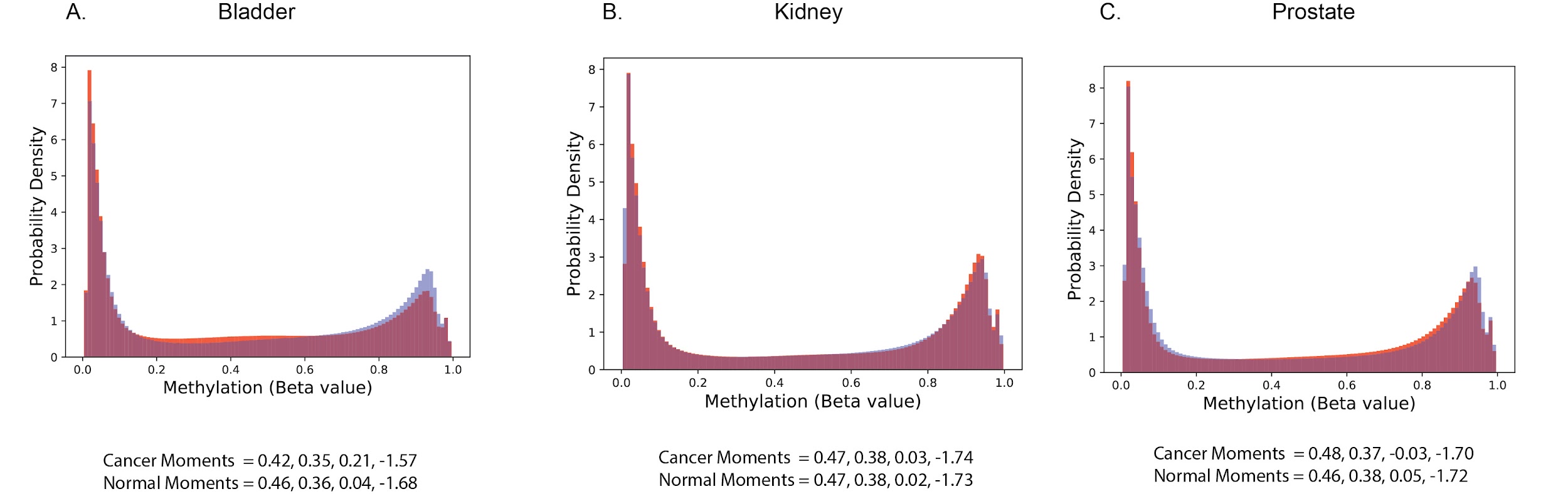


Following manifest files were uploaded to Zenodo and GitHub repositories: <https://doi.org/10.5281/zenodo.11530987> and <https://github.com/bazyliszek/methAI>

1. Bladder cancer manifest file: gdc_manifest.2019-11-09_bladder.txt
2. Kidney cancer manifest file: gdc_manifest.2019-11-09_kidney.txt
3. Prostate cancer manifest file: gdc_manifest.2019-10-19_prostate.txt
